# Supplementary material for: Nutrient Composition of Ovary, Hepatopancreas and Muscle Tissues in Relation to Ovarian Development Stage of Female Swimming Crab, Portunus trituberculatus
Source: Animals (Basel). 2023 Oct 15;13(20):3220. doi: 10.3390/ani13203220 (PMC10603639; doi:10.3390/ani13203220)
Supplement: Supplementary file 1 [file animals-13-03220-s001.zip › animals-2634710-supplementary.pdf]

Supplementary files

Supplementary Table S1. Factor loading, eigenvalue and variance explained by the 18 amino-acids from ovary in the principal component analysis.

	Component	
Amino acids	1	2
Thr	**0.94**	0.21
Cys	**0.92**	0.37
Met	**0.91**	0.30
Val	**0.91**	0.38
Ser	**0.89**	0.28
Gly	**0.88**	0.44
Pro	**0.88**	0.30
Tyr	**0.87**	0.44
Ala	**0.81**	0.53
Asp	**0.77**	0.44
Arg	**0.76**	0.61
Phe	**-0.74**	-0.21
Ile	**0.72**	0.66
Lys	**0.72**	0.67
His	**0.69**	0.48
Leu	**0.68**	0.61
Trp	0.08	**0.98**
Glu	0.44	**0.82**
Eigenvalue	14.73	1.31
Variance contribution rate (%)	81.83	7.30
Cumulative contribution rate (%)	81.83	89.12

Components with eigenvalues greater than 1.0 have been extracted to explain the variability of phenotypic traits. The loading scores greater than 0.65 and the highest compared to each of the other components are shown in bold.

Supplementary Table S2. Factor loading, eigenvalue and variance explained by the 18 amino-acids from hepatopancreas in the principal component analysis.

	Component	
Amino acids	1	2
Leu	**0.97**	0.15
Met	**0.93**	0.33
Ile	**0.91**	0.28
Ala	**0.88**	0.45
Phe	**0.84**	0.45
Asp	**0.84**	0.39
Tyr	**0.83**	0.51
Trp	**0.83**	0.37
Cys	**0.81**	0.48
His	**0.74**	0.62
Lys	**0.71**	0.61
Arg	**0.70**	0.69
Gly	0.15	**0.92**
Val	-0.39	**-0.85**
Pro	0.50	**0.81**
Glu	0.27	**0.81**
Ser	0.62	**0.75**
Thr	0.34	**0.74**
Eigenvalue	14.31	1.73
Variance contribution rate (%)	79.51	9.61
Cumulative contribution rate (%)	79.51	89.12

Components with eigenvalues greater than 1.0 have been extracted to explain the variability of phenotypic traits. The loading scores greater than 0.65 and the highest compared to each of the other components are shown in bold.

Supplementary Table S3. Factor loading, eigenvalue and variance explained by the 18 amino-acids from mussel in the principal component analysis.

	Component	
Amino acids	1	2
Asp	**0.93**	0.33
Glu	**0.90**	0.37
Ile	**0.89**	0.31
Cys	**0.87**	0.44
Val	**0.86**	0.35
Gly	**0.84**	0.46
Lys	**0.82**	0.52
Arg	**0.79**	0.59
Ala	**0.78**	0.54
Ser	**0.78**	0.55
Met	**0.76**	0.58
Thr	**0.75**	0.53
Pro	0.15	**0.80**
Tyr	0.48	**0.80**
Trp	0.48	**0.80**
Leu	0.64	**0.75**
His	0.57	**0.75**
Phe	0.55	**0.71**
Eigenvalue	15.00	1.07
Variance contribution rate (%)	83.34	5.93
Cumulative contribution rate (%)	83.34	89.26

Components with eigenvalues greater than 1.0 have been extracted to explain the variability of phenotypic traits. The loading scores greater than 0.65 and the highest compared to each of the other components are shown in bold.

Supplementary Table S4. Factor loading, eigenvalue and variance explained by the fatty acids from ovary in the principal component analysis.

	Component			
Phenotypic traits	1	2	3	4
C16:0	**0.971**	0.058	0.186	-0.076
SFA	**0.951**	-0.084	0.287	-0.007
C24:1	**-0.933**	-0.043	-0.164	-0.271
MUFA	**-0.930**	-0.112	-0.300	-0.161
C20:0	**0.881**	0.227	0.400	-0.013
C18:0	**0.876**	0.127	0.423	0.036
C20:3n-6	**0.866**	0.276	0.336	-0.071
C18:2n-6	**0.856**	0.425	0.211	-0.077
C14:0	**0.836**	0.029	-0.163	-0.375
C22:6n-3	**0.823**	0.286	0.440	-0.077
C18:1	**-0.804**	-0.178	-0.432	0.283
C20:2n-6	**0.749**	0.184	0.604	-0.010
C16:1	**-0.726**	-0.220	-0.515	-0.261
C22:0	**-0.720**	-0.668	-0.082	-0.040
C22:2n-6	**0.694**	0.011	0.632	-0.186
C18:3n-3	**-0.665**	0.204	0.011	-0.598
C22:1	**0.634**	0.343	-0.228	-0.014
n-3:n-6	0.054	**0.990**	0.073	-0.091
n-3HUFA	0.103	**0.990**	0.072	-0.001
C20:5n-3	0.171	**0.972**	0.059	0.083
HUFA	0.153	**0.738**	0.121	0.643
C14:1	0.176	-0.041	**0.830**	0.126
C18:3n-6	0.426	0.289	**0.777**	-0.199
n:6HUFA	0.128	0.133	0.110	**0.966**
C20:4n-6	-0.212	0.023	-0.092	**0.965**
C24:0	-0.632	-0.168	-0.303	**0.647**
Eigenvalue	15.28	4.05	3.250	1.690
Variance contribution rate (%)	58.79	15.59	12.480	6.510
Cumulative contribution rate (%)	58.79	74.38	86.860	93.370

Components with eigenvalues greater than 1.0 have been extracted to explain the variability of phenotypic traits. The loading scores greater than 0.65 and the highest compared to each of the other components are shown in bold.

Supplementary Table S5. Factor loading, eigenvalue and variance explained by the fatty acids from hepatopancreas in the principal component analysis.

	Component					
Phenotypic traits	1	2	3	4	5	6
C16:0	**-0.898**	0.041	-0.323	0.127	0.169	-0.109
C22:0	**0.867**	-0.079	-0.127	-0.219	0.101	0.045
C20:4n-6	**0.788**	-0.315	-0.194	-0.168	0.382	-0.001
n:6HUFA	**0.722**	0.111	0.574	-0.108	0.235	0.104
SFA	**-0.718**	0.491	-0.313	0.065	0.247	-0.244
C24:1	**0.709**	-0.252	0.323	-0.130	-0.460	0.186
HUFA	**0.655**	0.141	0.612	0.250	0.222	0.037
C18:0	-0.322	**0.891**	-0.077	0.052	-0.169	-0.064
C16:1	0.118	**-0.890**	0.098	-0.072	-0.033	0.138
C18:1	0.201	**-0.864**	-0.063	-0.119	-0.221	0.039
C20:2n-6	0.105	**0.703**	0.322	-0.097	-0.251	-0.126
MUFA	0.528	**-0.694**	0.180	-0.115	-0.352	0.163
C14:1	0.074	**0.646**	0.323	0.329	0.020	0.482
C18:2n-6	0.067	**0.638**	0.531	0.086	0.145	0.019
C22:1	0.461	**0.616**	0.247	0.288	0.350	-0.150
C18:3n-6	0.163	-0.086	**0.876**	0.129	0.159	0.168
C22:2n-6	-0.039	-0.001	**0.820**	-0.025	-0.390	0.108
C24:0	0.108	0.165	**0.776**	0.023	0.000	-0.052
n:3HUFA	-0.174	0.078	0.100	**0.952**	-0.033	-0.179
C20:5n-3	0.208	0.195	-0.029	**0.842**	-0.312	0.120
n-3:n-6	-0.531	-0.007	-0.094	**0.790**	-0.031	-0.252
C18:3n-3	-0.177	0.266	0.142	**0.741**	0.175	0.171
C22:6n-3	-0.221	-0.200	0.053	**0.699**	-0.123	-0.554
C14:0	0.059	0.067	0.055	-0.197	**0.915**	-0.031
C20:3n-6	0.098	-0.404	0.207	-0.122	-0.021	**0.770**
C20:0	-0.514	0.187	0.031	0.123	0.262	**-0.578**
Eigenvalue	8.14	5.79	3.440	2.170	1.77	1.26
Variance contribution					6.795	4.847
rate (%)	31.31	22.27	13.220	8.356		
Cumulative contribution rate (%)	31.31	53.58	66.800	75.158	81.953	86.8

Components with eigenvalues greater than 1.0 have been extracted to explain the variability of phenotypic traits. The loading scores greater than 0.65 and the highest compared to each of the other components are shown in bold.

Supplementary Table S6. Factor loading, eigenvalue and variance explained by the fatty acids from muscle in the principal component analysis.

	Component					
Phenotypic traits	1	2	3	4	5	6
HUFA	**0.968**	0.133	0.057	0.030	0.066	-0.104
C20:4n-6	**0.966**	0.135	-0.094	0.095	-0.030	-0.116
n:6HUFA	**0.963**	0.139	-0.065	0.029	0.045	-0.118
C14:0	**-0.884**	0.246	-0.317	0.080	0.080	-0.069
C16:1	**-0.882**	-0.226	0.142	-0.254	0.117	-0.185
C16:0	**0.651**	0.299	-0.483	0.426	0.160	-0.097
C18:2n-6	**-0.560**	-0.376	0.550	0.076	-0.118	-0.089
C18:0	0.218	**0.916**	-0.072	0.186	-0.143	0.146
SFA	0.138	**0.893**	-0.359	0.093	-0.017	0.026
C24:1	0.012	**-0.749**	0.075	-0.616	-0.059	0.099
MUFA	-0.623	**-0.733**	0.235	-0.085	-0.023	0.036
C20:0	0.561	**0.654**	0.078	0.197	-0.187	0.317
C14:1	-0.241	**0.648**	-0.456	0.499	0.000	-0.062
C20:3n-6	0.344	**0.569**	0.403	-0.094	0.511	-0.151
C24:0	0.469	**-0.568**	-0.259	-0.454	0.189	0.272
n-3HUFA	0.025	-0.051	**0.953**	0.010	0.162	0.116
n-3:n-6	-0.320	-0.094	**0.900**	0.028	0.143	0.126
C18:3n-3	0.233	-0.084	**0.855**	-0.085	0.261	0.037
C22:6n-3	0.004	-0.203	**0.748**	-0.112	-0.117	-0.501
C18:1	0.113	0.085	0.127	**0.933**	-0.043	0.175
C22:0	-0.271	-0.411	0.519	**-0.608**	-0.128	-0.156
C20:2n-6	-0.259	-0.289	0.150	**-0.581**	0.368	0.128
C18:3n-6	0.043	0.352	-0.097	0.026	**0.848**	-0.104
C22:1	0.186	0.304	-0.172	0.162	**-0.780**	-0.342
C22:2n-6	-0.045	0.308	-0.312	0.015	**-0.756**	0.065
C20:5n-3	-0.457	0.161	0.517	0.294	-0.027	**0.581**
Eigenvalue	9.66	5.19	3.934	2.475	1.483	1.059
Variance contribution rate (%)	37.16	19.96	15.132	9.521	5.705	4.072
Cumulative contribution rate (%)	37.16	57.12	72.253	81.774	87.479	91.551

Components with eigenvalues greater than 1.0 have been extracted to explain the variability of phenotypic traits. The loading scores greater than 0.65 and the highest compared to each of the other components are shown in bold.
